# Effect of different intra-canal restorative techniques on the fracture resistance of endodontically treated anterior teeth: an in-vitro comparative study

**DOI:** 10.1186/s12903-026-09837-8

**Published:** 2026-09-30

**Authors:** Mohammed Ehab Mohammed Ali Saad, Nahla Hamed Mohamed Hamed, Mohammed Nasser Anwar

**Affiliations:** 1 Department of Operative Dentistry, Faculty of Dentistry, New Mansoura University, Mansoura, Egypt; 2 Department of Operative Dentistry, Faculty of Dentistry, Ain Shams University, Cairo, Egypt

**Keywords:** Endodontically treated anterior teeth, Fiber posts, Short fiber‑reinforced composite, Fracture resistance, Intra-canal, Fracture pattern

## Abstract

**Objective:**

To evaluate the effect of different intra‑canal restorative techniques on the fracture resistance of endodontically treated anterior teeth and to assess the influence of marginal ridge loss on their mechanical behavior.

**Materials and methods:**

Ninety extracted sound maxillary central and lateral incisors were selected and randomly divided into five groups. Ten teeth were left intact to serve as the control group, while the remaining teeth were endodontically treated, obturated, and randomly allocated into four groups according to different intra‑canal restorative techniques; (1) PS: packable short fiber‑reinforced composite, (2) FP: prefabricated glass fiber posts, (3) FS: flowable short fiber‑reinforced composite, (4) DC: dual‑cure composite core material, each group was further subdivided according to marginal ridge integrity into two subgroups (*n* = 10), intact marginal ridges (I) and removed marginal ridge (R). All specimens were subjected to thermocycling for 10,000 cycles. Fracture resistance testing was carried out using a universal testing machine with a load applied at 135° to the long axis of the tooth until the fracture occurred. Fracture patterns were examined using a stereomicroscope and classified as favorable and unfavorable fractures. The data were statistically analyzed using two-way ANOVA, followed by Tukey’s post hoc test and fracture patterns were analyzed using Fisher’s exact test, with the significance level set at α = 0.05.

**Results:**

Intra-canal restorative technique and marginal ridge integrity significantly affected fracture resistance (*p* < 0.05), whereas no significant interaction was observed (*p* = 0.564). Fiber post restorations demonstrated the highest fracture resistance values. No statistically significant differences were observed among fiber posts, packable short fiber-reinforced composite, and flowable short fiber-reinforced composite under either marginal ridge condition (*p* > 0.05). Loss of one marginal ridge significantly reduced fracture resistance across all restorative techniques. The fracture pattern was significantly associated with the restorative technique (*p* = 0.014), with flowable short fiber-reinforced composite showing the highest proportion of favorable fractures.

**Conclusions:**

SFRC intra-canal restorations showed fracture resistance values that were not statistically different from those of fiber post restorations.

Marginal ridge loss significantly compromised fracture resistance of endodontically treated anterior teeth.

**Clinical significance:**

SFRC may represent a promising post-free restorative option for endodontically treated anterior teeth when sufficient coronal tooth structure remains, particularly in teeth with preserved marginal ridge integrity. However, long-term clinical studies and fatigue-loading investigations are required before definitive clinical recommendations can be made.

## Background

Due to the loss of tooth structure by caries, trauma, and endodontic procedures, endodontically treated teeth are more prone to fracture than vital teeth. Dentin removal during root canal instrumentation and access cavity preparation may drastically decrease tooth stiffness and jeopardize structural integrity. Consequently, the restoration of endodontically treated teeth remains a major challenge in restorative dentistry, [1, 2] especially in anterior teeth which are subjected to tensile functional stresses during protrusive and lateral mandibular movements, rather than purely compressive loads [3]. These loading patterns increases the risk of fracture. Therefore, selection of the optimum post-endodontic restorative strategy remains controversial in these clinical scenarios.

Glass fiber posts have traditionally been used for restoring endodontically treated teeth, particularly when substantial coronal tooth structure has been lost. It was stated that fiber posts could potentially increase the fracture resistance of endodontically treated teeth [4], but recently several studies did not find an increase in fracture resistance when inserted with composite restorations. Stating that the role of root canal posts may act as a retentive system for the core rather than as a reinforcing element [5]. Despite the clinical success of using fiber posts, the greatest disadvantage is the additional removal of dentin required to insert the post into the root canal. Furthermore, this approach has been demonstrated to affect the overall biomechanical behavior of restored teeth. As a result, the additional retention obtained from a post must be balanced against the loss of healthy tissues, which eventually weakens the tooth [6].

Short fiber-reinforced composites SFRC have been proposed for biomimetic replacement of dentin in larger cavities and endodontically treated teeth. Manufacturers claim that short fiber-reinforced composites strengthen restorations by increasing fracture toughness and inhibiting crack propagation [7]. This occurs due to stress transfer from the polymer matrix to the fibers [7]. According to various authors, the traditional method of using fiber post is more complex and sensitive than using SFRC as a post-core foundation [7].

Comparative data between SFRCs and fiber glass posts in terms of post-endodontic reinforcement, specifically in anterior teeth with standardized endodontic treatment protocols, remains limited. Furthermore, most existing research focuses on posterior teeth or uses various methodologies, making it difficult to draw firm conclusions. There is a significant need for well-controlled studies that directly evaluate the biomechanical performance of SFRCs and fiber glass posts in endodontically treated anterior teeth which are subjected to different loading patterns. Although short fiber-reinforced composites have demonstrated promising mechanical properties, evidence regarding their intra-canal application in endodontically treated anterior teeth, particularly in relation to marginal ridge integrity, remains limited. Therefore, further investigation is warranted to evaluate their fracture resistance and fracture behavior under these clinical conditions. The purpose of this study was to evaluate the fracture resistance and the fracture pattern of different intra-canal restorative techniques in endodontically treated anterior teeth and how the marginal ridge integrity could affect their mechanical behavior.

The null hypothesis was:

- There is no difference in fracture resistance among endodontically treated anterior teeth restored with different intra-canal restorative techniques.
- Loss of one marginal ridge does not affect the fracture resistance of endodontically treated anterior teeth restored using different intra-canal restorative techniques.

## Materials and methods

The materials used in this study are presented in (Table 1). Ninety extracted human maxillary incisors, comprising 49 central incisors and 41 lateral incisors extracted for periodontal reasons, were included in this study. Teeth were first stratified according to tooth type and then randomly allocated to the experimental groups using a simple manual randomization method to ensure a balanced distribution of central and lateral incisors across all groups. The inclusion criteria were absence of caries, cervical abrasion, abfraction and crack lines. Teeth were selected by measuring the apicoincisal length, labiopalatal, and mesiodistal widths (in mm), permitting a maximum deviation of 10% from the average, using a manual caliper [8]. Radiographic evaluation of each tooth was performed to ensure that no internal resorption or obstruction existed within the canal system. Calculus and periodontal tissue remnants were removed with an ultrasonic scaler and stored in distilled water at room temperature during the study.

**Table 1:** Materials (Lot #), composition and manufacturer used in the study

Materials	Description	Composition	Manufacturer	Lot #
Neo Spectra ST LV	Universal nanohybrid resin composite Shade: A2	Spherical pre-polymerized SphereTEC fillers, non-agglomerated barium glass and ytterbium fluoride, highly dispersed methacrylic polysiloxane nano-particles	Dentsply Sirona GmbH, Konstanz, Germany	2308000623
Vislays Cemcore	Dual-cure adhesive resin cement and core build-up composite Shade: universal A2	UDMA, other Dimethacrylate (aliphatic Trimethacrylate / aliphatic Dimethacrylate), Ytterbium fluoride and silica Polymorph, Benzoyl peroxide.	Kettenbach GmbH & Co. KG, Eschenburg, Germany	232411115
EverX Posterior	Packable SFRC Shade: bulk shade	Bis-GMA, PMMA, TEGDMA, millimetre scale glass fiber filler, Barium glass 76 wt%, 57 vol%.	GC, Tokyo, Japan	2311071
EverX Flow	flowable SFRC shade: bulk shade	Bis-EMA, TEGDMA, UDMA, micrometer scale glass fiber filler, Barium glass 70 wt %, 46 vol %	GC, Tokyo, Japan	2202011
ParaBond	Chemical cured adhesive ParaBond adhesive A ParaBond adhesive B	Methacrylates, Maleic acid, Benzoyl peroxide. Ethanol, Water, Initiators	Coltène, Whaledent, Altstätten, Switzerland	M72688
Avant Bioloren	Prefabricated glass fiber posts	Epoxy resin matrix (25%vol), multidirectional glass fiber reinforcement (75% vol)	Bioloren, saronno, Italy	23\24

### Sample size calculation

Sample size calculation was performed using R statistical software (version 4.3.2 for Windows) based on the fracture resistance results reported in a previous study [1]. Assuming an effect size (f) of 0.622, a significance level (α) of 0.05, and a statistical power of 80% (β = 0.20), the minimum required sample size was calculated to be 40 specimens (8 specimens per group). To increase the reliability of the findings, 10 specimens were included in each experimental subgroup.

### Access cavity preparation

Ten teeth were left intact and served as positive control group (C). In the remaining 80 specimens, a conventional access cavity was prepared using a small blue coded round diamond stone (Komet, Trophagener Weg, Germany) with a high-speed air turbine water-cooled handpiece (NSK PANA AIR FX, Nakanishi Inc., Shimohinata Kanuma, Japan) through the center of the palatal surface above the cingulum. Pulp horns were removed in the mesio-distal direction using a tapered blue coded diamond stone ISO 199/016-TR 12.

### Root canal treatment

**Fig. 1:**
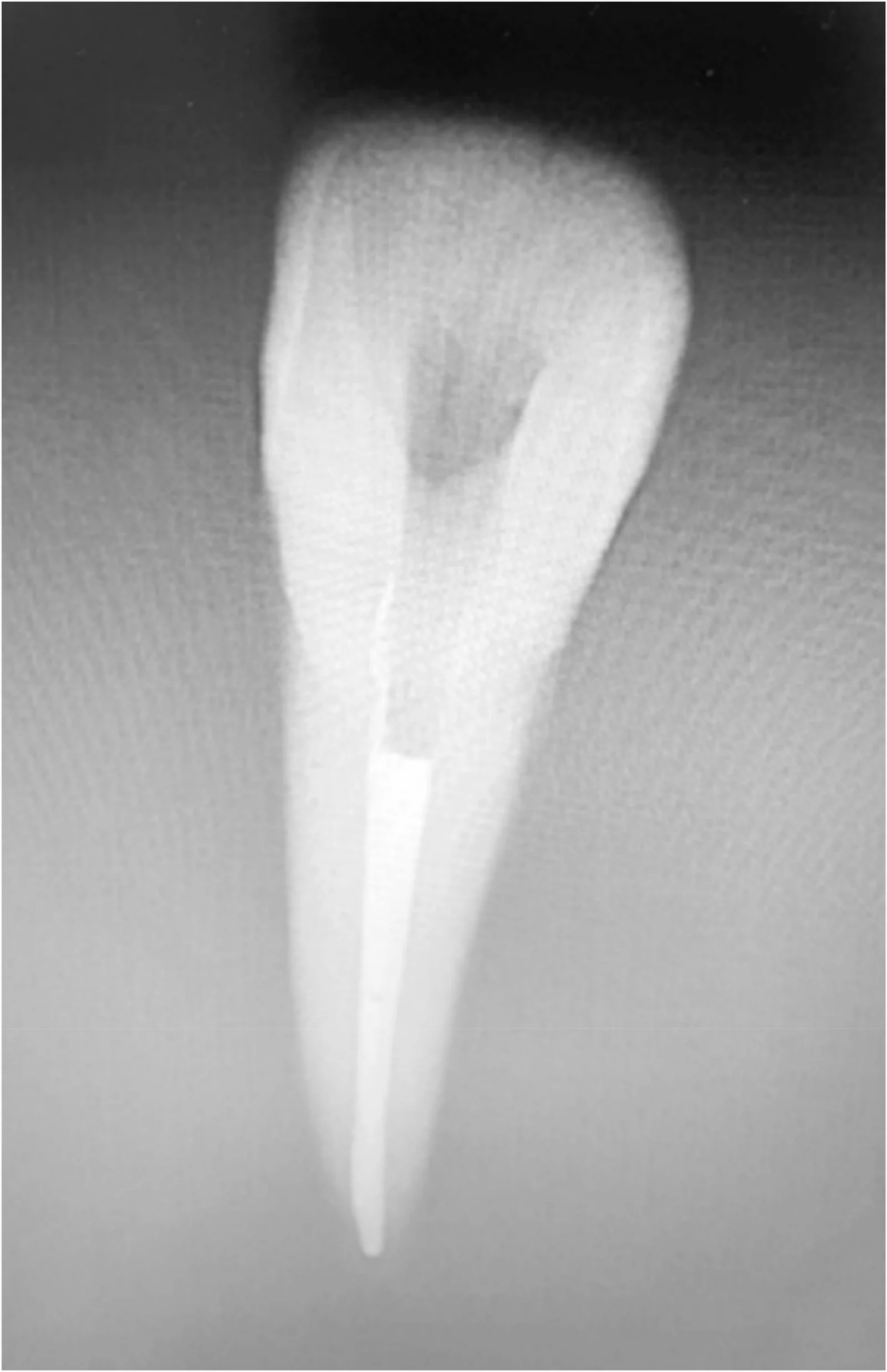
Radiographic confirmation of gutta-percha removal to 4 mm below the CEJ

The ISO #10 size K-file was inserted into the root canal. The working length was determined radiographically and was set to be 1 mm less than the measured canal length. The root canals were prepared to a size 40 taper 4 with the Fanta blue rotary instrumentation system (FANTA DENTAL, SHANGHAI, China). Before the insertion of each file, the canals were irrigated with 2 mL of 2.5% sodium hypochlorite (NaOCl). The final irrigation involved 2 mL of 17% ethylenediaminetetraacetic acid (EDTA), accompanied by 2 mL of 2.5% sodium hypochlorite (NaOCl) separated by saline. The canals were dried using paper points (Meta Biomed Co, Ltd) to remove the chemical residues of the irrigation solutions, then the canals were filled with a single size 40 taper 4 gutta percha cone with a eugenol-free epoxyamine resin sealer (ADSEAL; Meta Biomed, Incheon, Korea). The gutta percha was removed to a depth of 4 mm from the cemento-enamel junction using a heated plugger (EQ-V Pack, Meta Biomed, Incheon, Korea) [1]. Radiographic evaluation was used for confirmation (Fig. 1). 

**Fig. 2:**
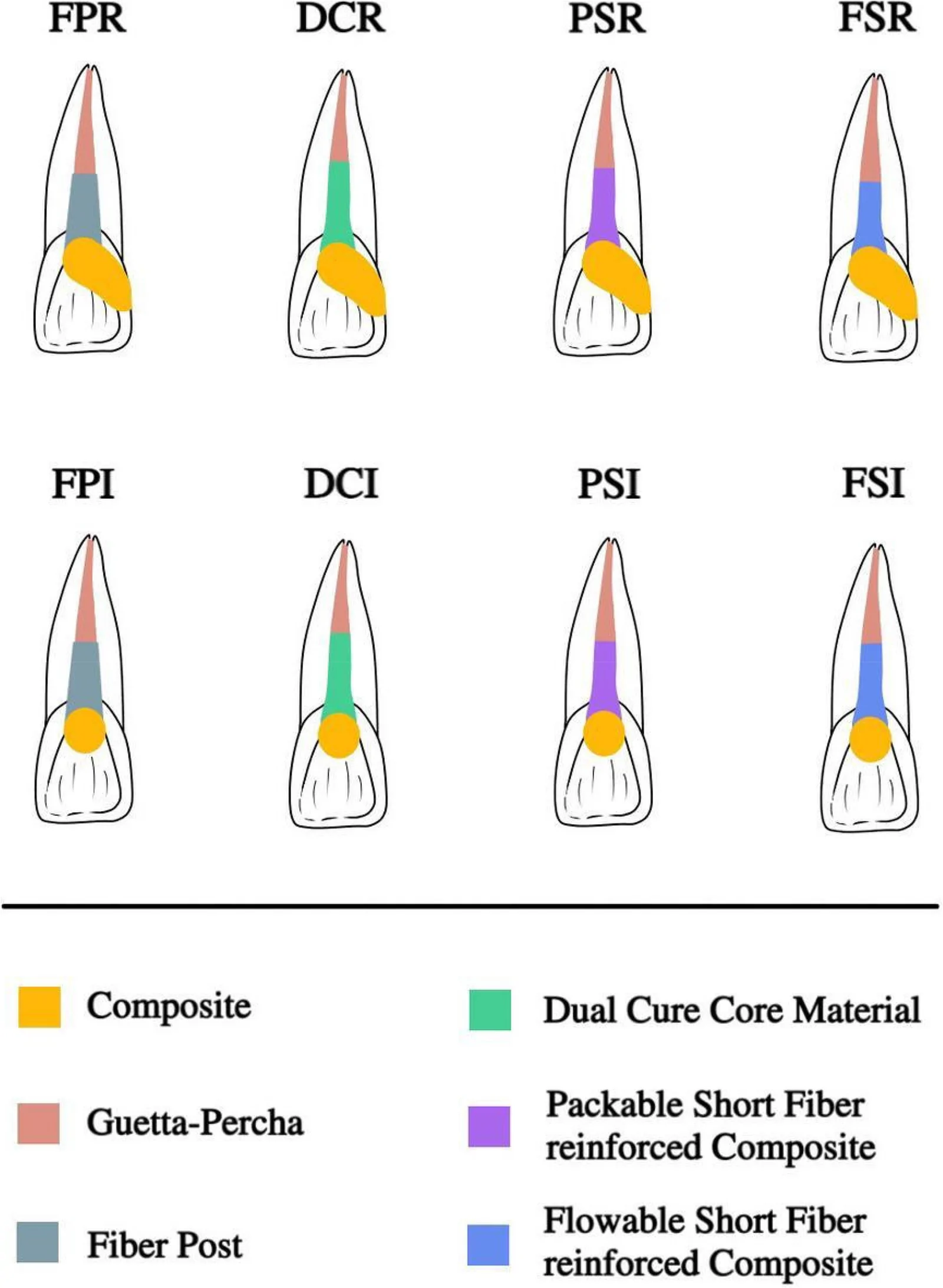
Schematic of experimental groups by intra-canal restorative technique (FP, DC, PS, FS) and marginal ridge condition (R, removed; I, intact), with color coding indicating materials used

The teeth were randomly categorized into 4 groups of 20 teeth each according to the following intra-canal restorative techniques (Fig. 2): 

1. Packable short fiber reinforced composite (PS).
2. Fiber post (FP).
3. Flowable short fiber reinforced composite (FS).
4. Dual cure composite core material (DC).

Each was further subdivided into two groups of 10 teeth each, based on marginal ridge condition into the following: intact marginal ridge (I) and removed marginal ridge (R).

### Proximal cavity preparations

**Fig. 3:**
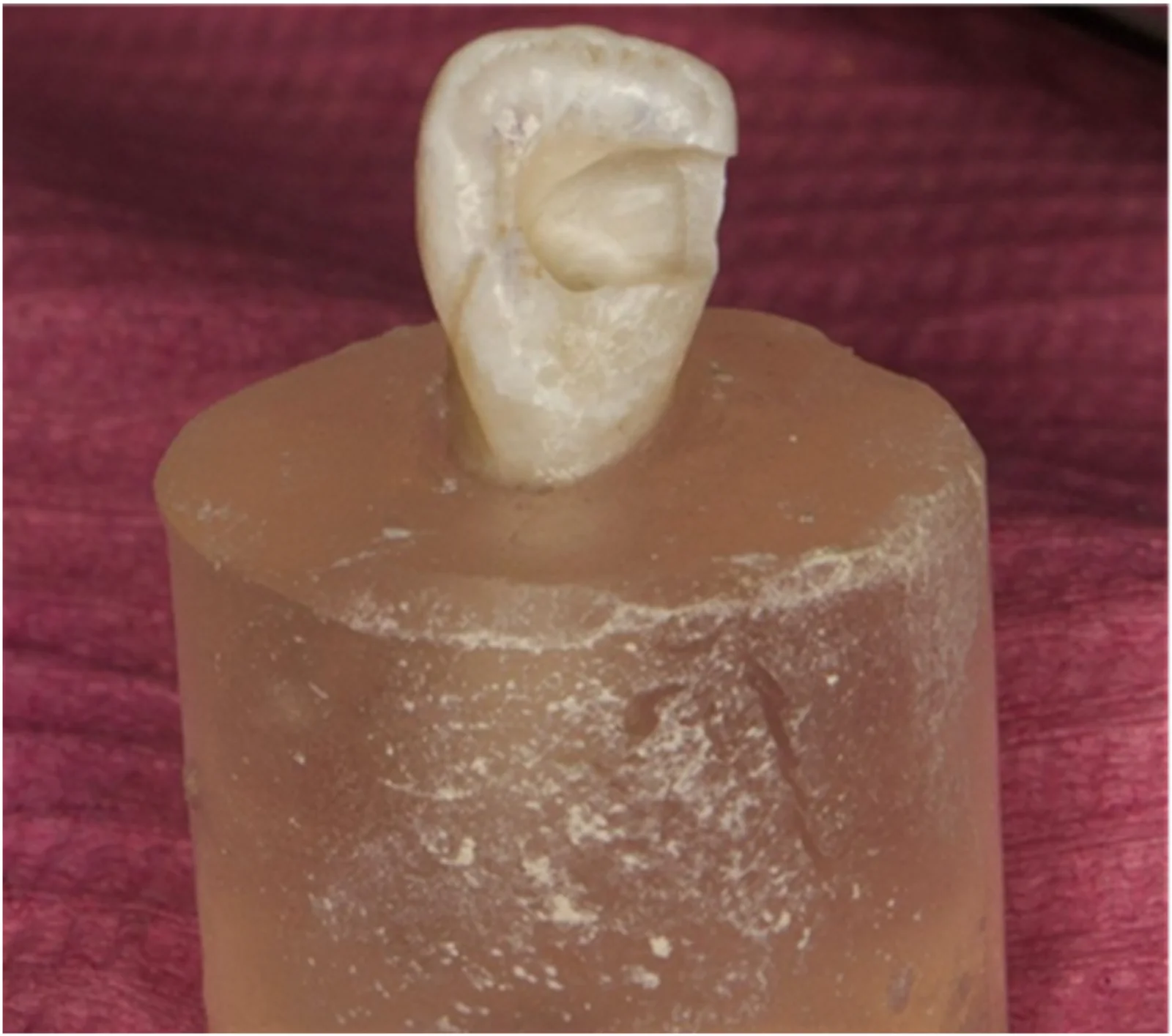
Proximal cavity preparation

In the subgroups PSR, FPR, FSR, and DCR, a class III cavity was prepared on the mesial surface using a 330 bur (Komet, USA). To maximize the reproducibility of cavity dimensions, the mesio-distal, linguo-labial, and cervical-incisal extents of the crowns were measured with a caliper. Class III cavities included one-third of the mesio-distal and linguo-labial dimensions and one-third of the cervical-incisal region (Fig. 3). The median part of the cavity was always connected to the endodontic access cavities [1]. 

### Embedding the teeth specimens in acrylic cylinders

To ensure standardized positioning, each tooth specimen was mounted using a dental surveyor, aligning its long axis perpendicular to the base of the mold. The roots were then embedded in auto-polymerizing acrylic resin (Imicryl, Konya, Turkey) up to 1 mm apical to the cemento-enamel junction. The resin was mixed according to the manufacturer’s instructions and placed in a plastic tube mold. The vertical arm of the surveyor pin fixator was adjusted to position the tooth accurately within the acrylic resin, after which the specimen was stabilized by tightening the fixing screw. Each specimen was maintained in this standardized position until complete polymerization [9]. 

### Surface treatment

Selective enamel etching was done using a 37% phosphoric acid etchant for 30 s (Meta Etchant; Meta Biomed), followed by a 30-second water spray rinse and gentle air drying. Parabond was incorporated in a 1:1 ratio of chemically cured adhesive A and B (Coltène/Whaledent GmbH, Altstätten, Switzerland) and applied for 30 s on the cavity dentin, root canal and enamel margins. The excess adhesive was removed with paper points and air-dried for 5 s, in compliance with the manufacturer’s recommendations [10]. 

### Intra-canal restorative techniques

#### Group 1: packable short fiber reinforced composite

A 1 mm layer of EverX Flow was injected intra-canal to facilitate the packing and adaption of EverX posterior (GC, Tokyo, Japan), which was performed incrementally in 2 mm thickness using a micro-brush to a maximum intra-canal depth of 4 mm below the CEJ [11]. Before polymerization, the output intensity of the LED curing unit was verified using a radiometer and measured 1200 mW/cm² [12]. The light guide tip was positioned directly over the canal opening during light curing to standardize light delivery. Each layer was individually cured for an extended duration of 40 s using the Bluephase (Ivoclar Vivadent Schaan, Liechtenstein) LED light-curing device. The root canal was filled to the level of the CEJ, then applied in layers up to 2 mm below the access cavity margins, light-cured, and subsequently covered with a universal nanohybrid restorative composite (Neo Spectra ST LV, DENTSPLY Sirona, Konstanz, Germany). Finishing and contouring were performed using a yellow-coded diamond stone, followed by final polishing with a two-step diamond-impregnated polishing discs (EVE, Pforzheim, Germany).

#### Group 2: flowable short fiber reinforced composite

A 2 mm initial increment of EverX Flow was injected intra-canal incrementally to ensure full depth of cure; each layer was light cured separately for 40 s. The canal was filled up to 2 mm below the access cavity margins and cured for 40 s, [11] the access cavity was restored with a restorative nanohybrid composite resin, light cured for 20 s, then finished and polished as mentioned before.

#### Group 3: fiber post

Prefabricated glass fiber posts with a diameter of 1.2–1.65 mm (AVANT, Bioloren, Italy) were trial-fitted within the canal, cut to a length 1 mm under the margins of the access cavity, and subsequently cleansed with alcohol. Silane coupling agent (Bisco, Schaumburg, IL, USA) was applied only to the fiber posts and air dried for 5 s, subsequently coated with freshly mixed self-adhesive dual-cure resin cement (vislays cemcore, Kettenbach, Eschenburg, Germany) and injected into the root canal. Each post was seated with finger pressure for 10 s and then light cured for 20 s. The access cavity was restored with a universal nanohybrid composite resin and subsequently refined and polished as previously described [1].

#### Group 4: dual cure composite core material

Vislays cemcore was injected intra-canal with designated tips for easier injection. The canal was filled up to 2 mm below the access cavity margins, then light cured for 20 s, complete polymerization was achieved through the material’s dual-cure mechanism according to the manufacturer’s instructions, then capped with nano-hybrid restorative composite resin.

**Fig. 4:**
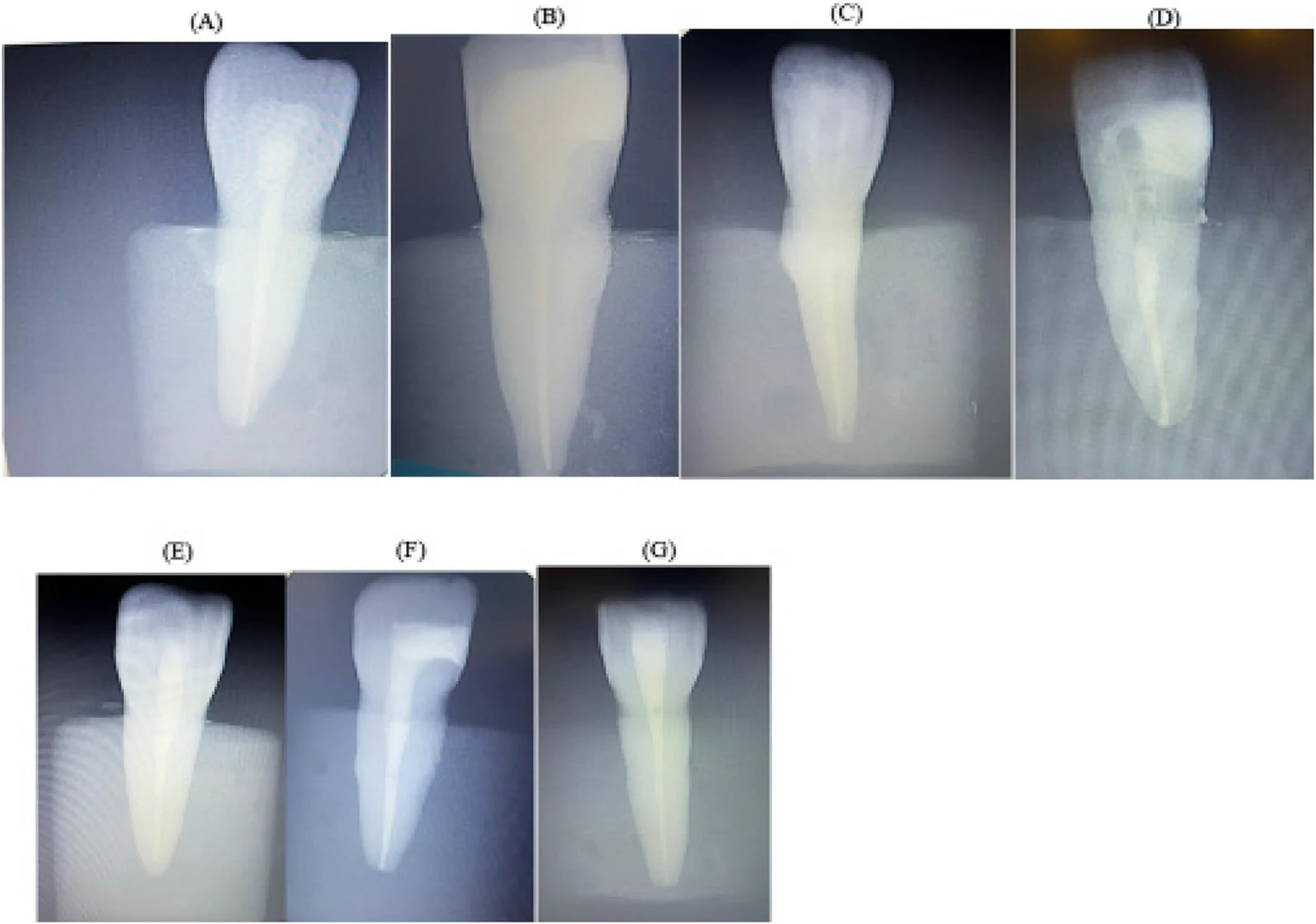
Post-operative radiographs of representative specimens from the different study groups. **A **PSI. **B** PSR. **C** FPI. **D** FPR. **E** FSI. **F** FSR. **G **DCI

The proximal cavities of subgroups PSR, FPR, FSR and DCR were restored by a universal nano-hybrid composite resin (NeoSpectra ST LV, DENTSPLY Sirona, Konstanz, Germany), layered in a 2 mm thickness, each layer was light cured for 20 s, then finished and polished as mentioned before. Post-operative X-rays of different study groups are represented in (Fig. 4).

### Thermocycling

Specimens were thermocycled for 10,000 cycles, a commonly used artificial aging protocol intended to simulate thermal stresses in the oral environment [13]. Thermocycling was performed using an SD Mechatronik thermocycler (SD Mechatronik GmbH, Feldkirchen-Westerham, Germany) between 5 °C and 55 °C. Each cycle consisted of a 30-second dwell time in each water bath with a 10-second transfer time between baths. Before fracture resistance testing, the specimens were stored in saline solution at 37 °C and 100% humidity for one week [14].

### Fracture resistance testing

**Fig. 5:**
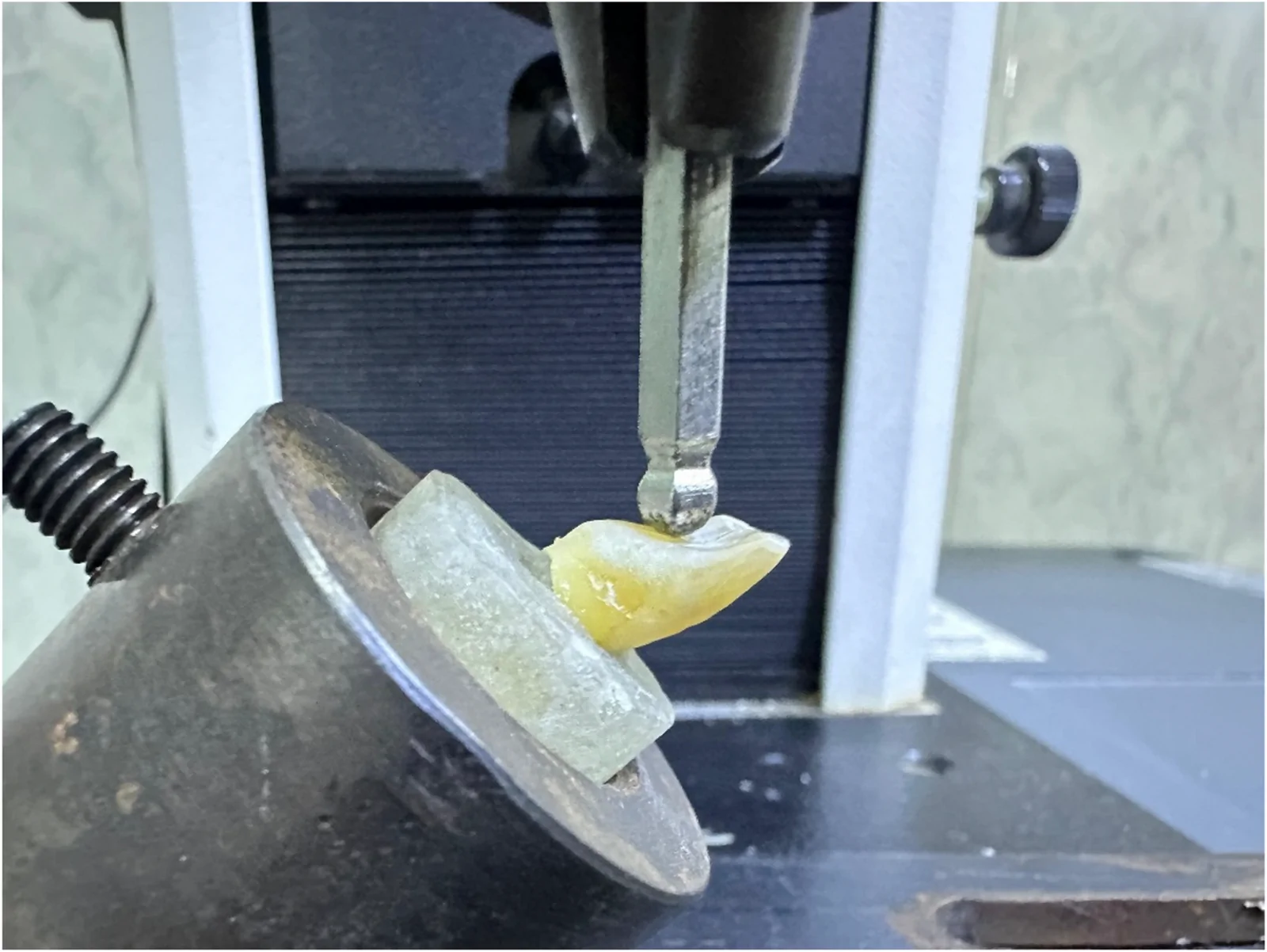
Fracture resistance testing.

Prior to fracture resistance testing, all specimens were assigned coded identifiers. Consequently, the operator performing the mechanical testing was blinded to the group allocation throughout the evaluation. All specimens were tested using the INSTRON type 3345 universal testing machine. The load was applied to the mid-palatal region of the crown, 3 mm from the incisal edge, at an angle of 135 degrees to the long axis of the root (Fig. 5), using a steel rod with a rounded tip and a diameter of 2 mm, at an average speed of 1 mm/min until fracture, [15] to simulate the oblique forces exerted on maxillary incisors during functional and parafunctional movements. Fracture resistance values were recorded and documented in newtons utilizing computer software (BlueHill Universal Instron, England). The fracture pattern of the specimens was assessed utilizing an optical and digital stereo microscope (XCAM 1080P8MPB, ToupTek Photonics, Zhejiang, China), distinguishing between favorable fractures (restorable) above the cemento-enamel junction and unfavorable fractures (non-restorable) below the cemento-enamel junction. The examiner assessing fracture patterns was blinded to the group allocation throughout the evaluation.

### Statistical analysis

The collected data were analyzed for normal distribution using Shapiro–Wilk test. The P values showed that all groups were normally distributed (*P* > 0.05). Two-way ANOVA was used to evaluate the effect of intra-canal restorative technique and marginal ridge condition, as well as their interaction. When significant differences were detected, post hoc pairwise comparisons were performed using Tukey’s test. Fracture pattern data were analyzed using Fisher’s exact test to assess the association between fracture pattern distribution and the eight experimental subgroups according to the restorative technique. The intact control group was excluded from this analysis because it did not belong to either marginal ridge condition and is presented descriptively for reference only. The level of statistical significance was set at *p* < 0.05. Data were analyzed using the Statistical Package for Social Science (SPSS Inc., Chicago, IL, USA; version 20.0).

## Results

**Fig. 6:**
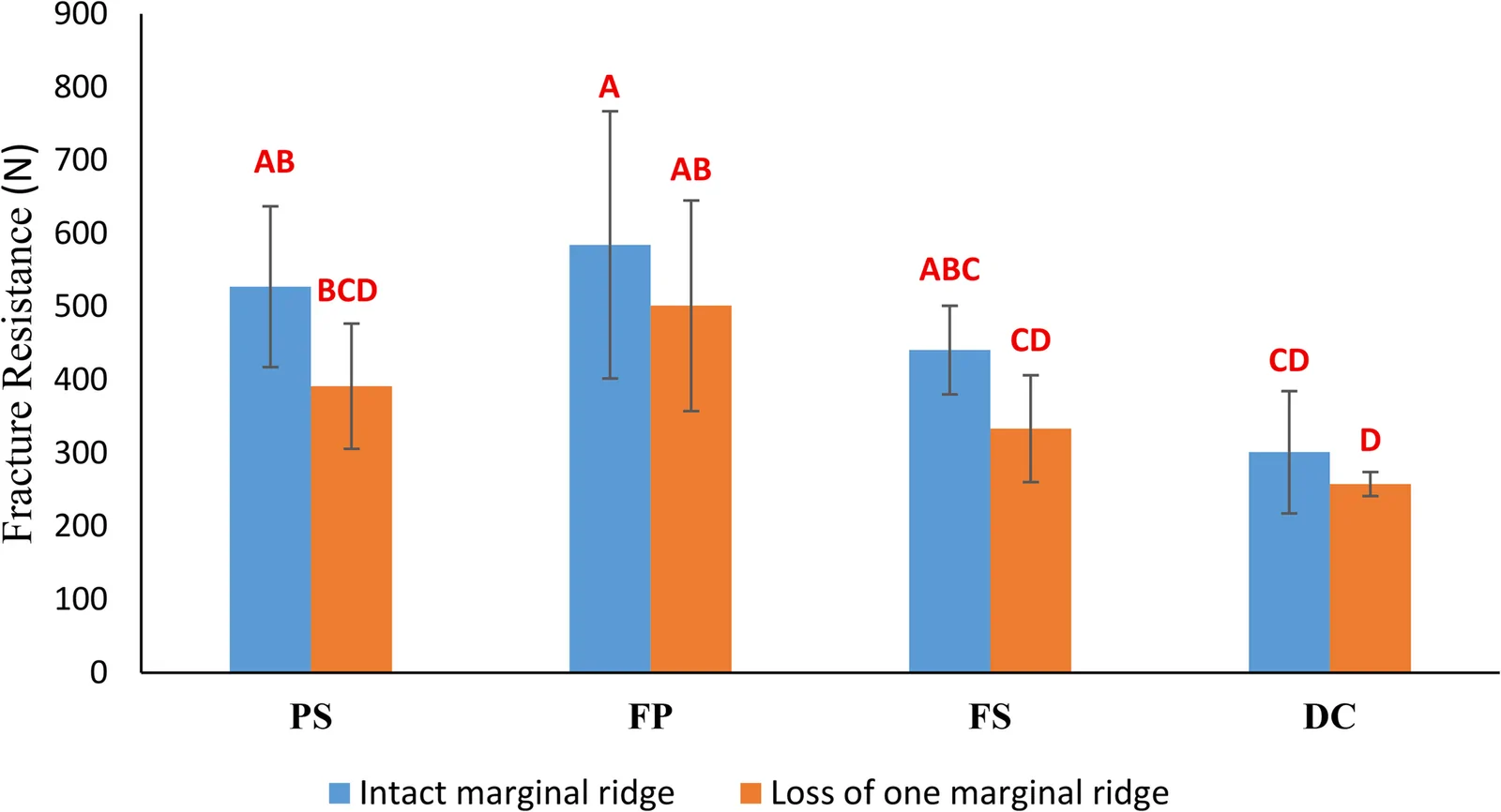
Bar chart representing the mean fracture resistance among different study groups. (Intact control group was omitted from the leftt because it served as a reference group and was not included in the factorial analysis)

Two-way ANOVA revealed statistically significant main effects of the intra-canal restorative technique and marginal ridge integrity (*p* < 0.001) on fracture resistance, whereas no statistically significant interaction was observed between the two factors (*p* = 0.564). The corresponding ANOVA results, including effect size measures, are presented in (Table 2), while descriptive statistics (means, standard deviations, and 95% confidence intervals) are presented in (Table 3). Descriptive fracture resistance data are also illustrated in (Fig. 6). Fiber post restorations demonstrated the highest fracture resistance values, whereas the dual-cure composite core material showed the lowest values. Tukey’s post hoc analysis demonstrated no significant differences among the fiber post, packable SFRC, and flowable SFRC groups under either marginal ridge condition (*p* > 0.05). In contrast, fiber post restorations exhibited significantly higher fracture resistance than the dual-cure composite group under both intact (*p* < 0.001) and removed (*p* < 0.001) marginal ridge conditions. Marginal ridge loss significantly reduced fracture resistance regardless of the intra-canal restorative technique (Table 2).

**Table 2:** Two-way ANOVA for fracture resistance according to intra-canal restorative technique and marginal ridge integrity, including partial eta squared (η²) effect sizes

Source	Sum of squares	df	Mean square	F-value	*P*-value	Partial η²
Corrected Model	942150.557a	7	134592.937	12.023	< 0.001*	0.539
Intercept	13922343.733	1	13922343.733	1243.653	< 0.001*	0.945
Intra-canal restorative technique	748276.163	3	249425.388	22.281	< 0.001*	0.481
Marginal ridge integrity	170867.484	1	170867.484	15.263	< 0.001*	0.175
Intra-canal restorative technique * Marginal ridge integrity	23006.910	3	7668.970	0.685	0.564	0.028
Error	806019.863	72	11194.720			
Total	15670514.153	80				
Corrected Total	1748170.420	79				

*Statistically significant difference at *P* < 0.05

**Table 3:** Mean fracture resistance (N), standard deviation, and 95% confidence intervals for the experimental groups

Type of intra-canal restorative technique	Marginal ridge integrity	95% Confidence Interval for Mean		Mean ± SD (*N*)
		Lower Bound (*N*)	Upper Bound (*N*)	
Control (C)	-	797.4852	1178.8968	988.19 ± 266.58
Short fiber reinforced packable composite (PS)	PSI	448.7811	605.7829	527.28 ± 109.73^AB^
	PSR	330.2114	452.5286	391.37 ± 85.49^BCD^
Fiber post (FP)	FPI	453.7322	714.8388	584.28 ± 182.50^A^
	FPR	398.4394	604.1566	501.29 ± 143.78^AB^
Short fiber reinforced flowable composite (FS)	FSI	397.4966	483.9414	440.71 ± 60.42^ABC^
	FSR	281.1332	385.5728	333.35 ± 72.99^CD^
Core material (DC)	DCI	241.4962	360.9978	301.24 ± 83.52^CD^
	DCR	246.0508	269.5312	257.79 ± 16.41^D^

Means sharing the same superscript letter are not significantly different according to Tukey’s post hoc test at *p* > 0.05

The Control group was excluded from the two-way ANOVA and Tukey post-hoc comparisons and presented for descriptive purposes only

I: intact marginal ridge

R: removed marginal ridge

**Fig. 7:**
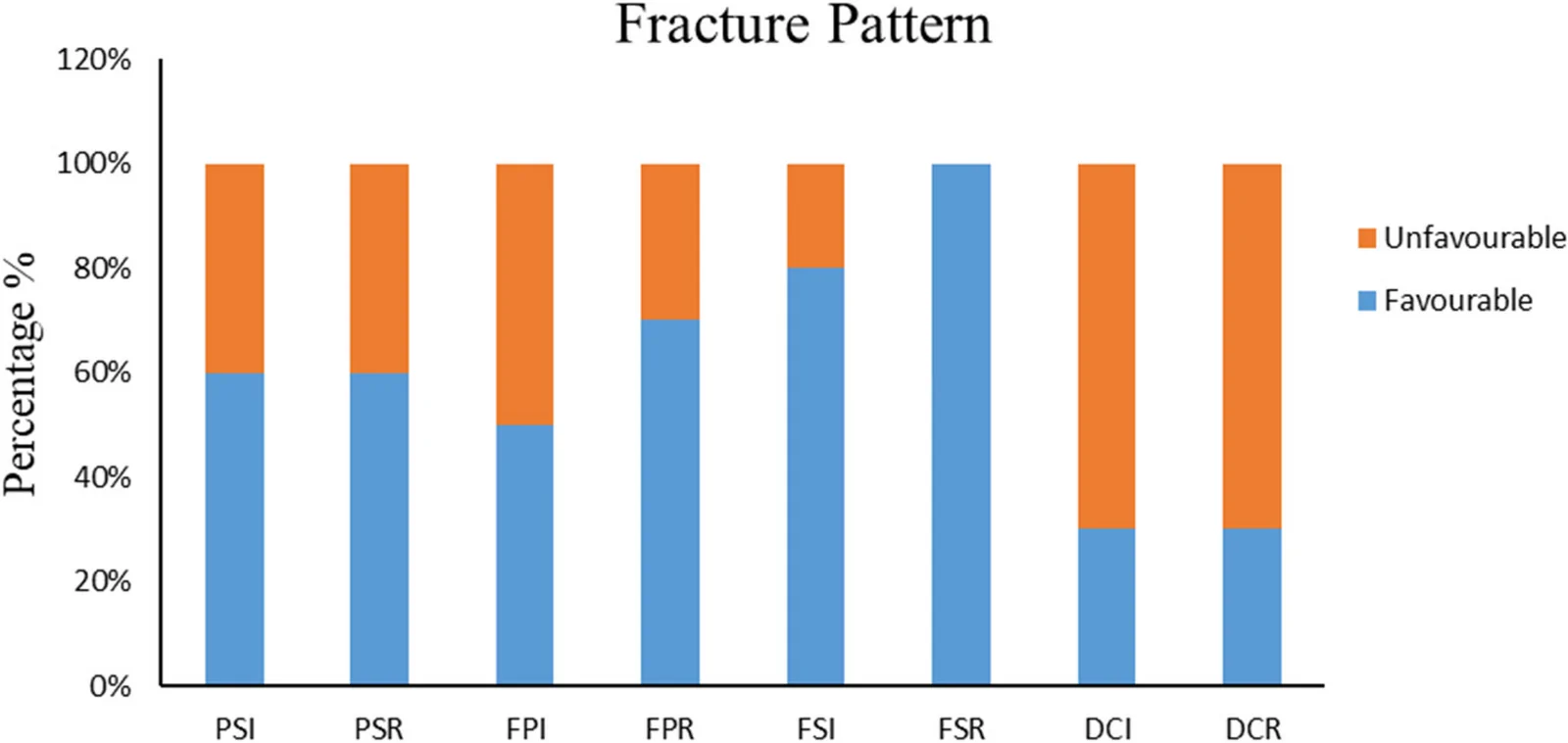
Bar chart representing the percentage distribution of fracture patterns among study groups. (I: intact marginal ridges, R: removed marginal ridge)

**Fig. 8:**
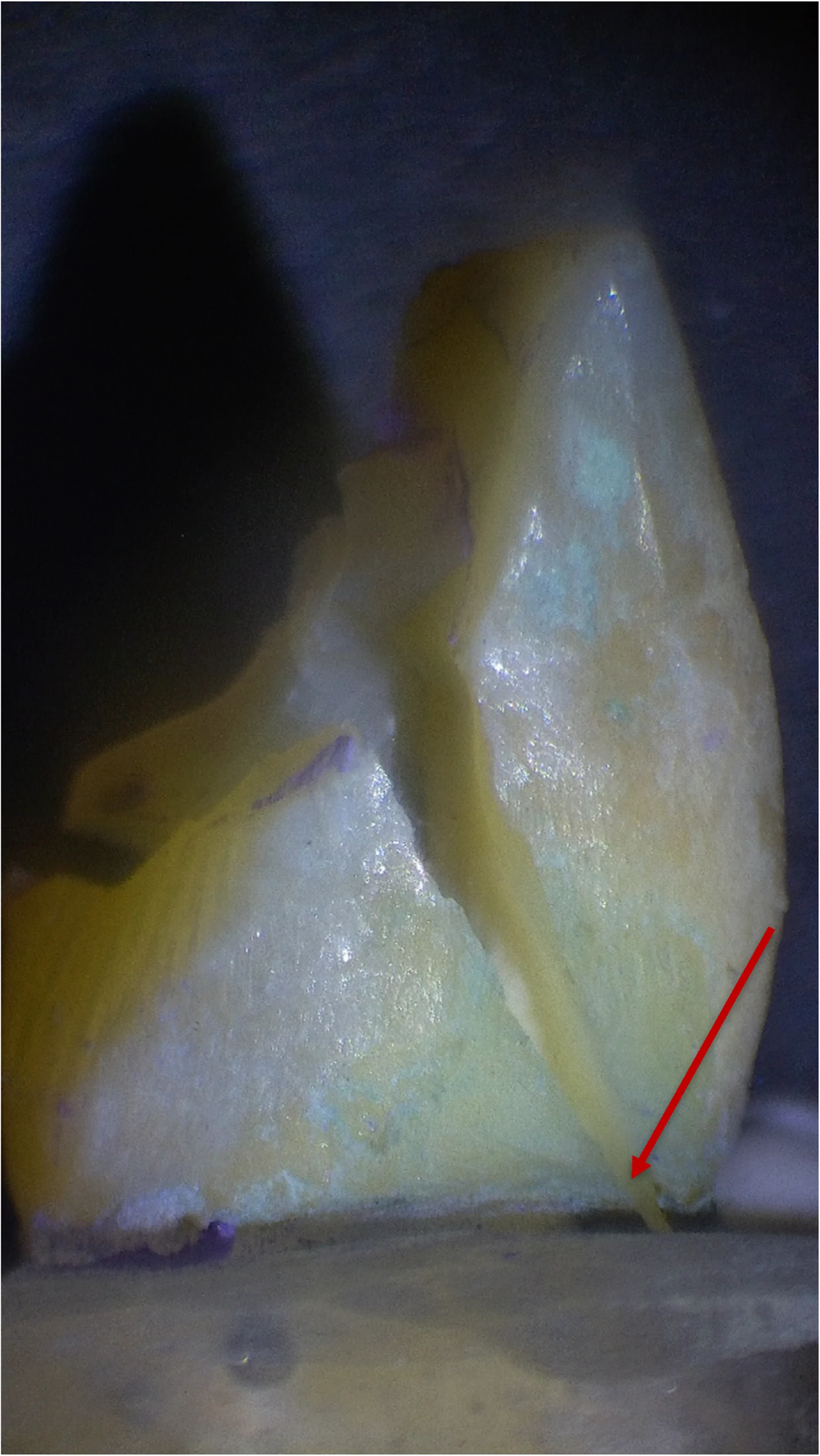
A stereomicroscope image with 8× magnification showing an unfavorable fracture pattern (below the CEJ). Arrow indicating fracture site

**Fig. 9:**
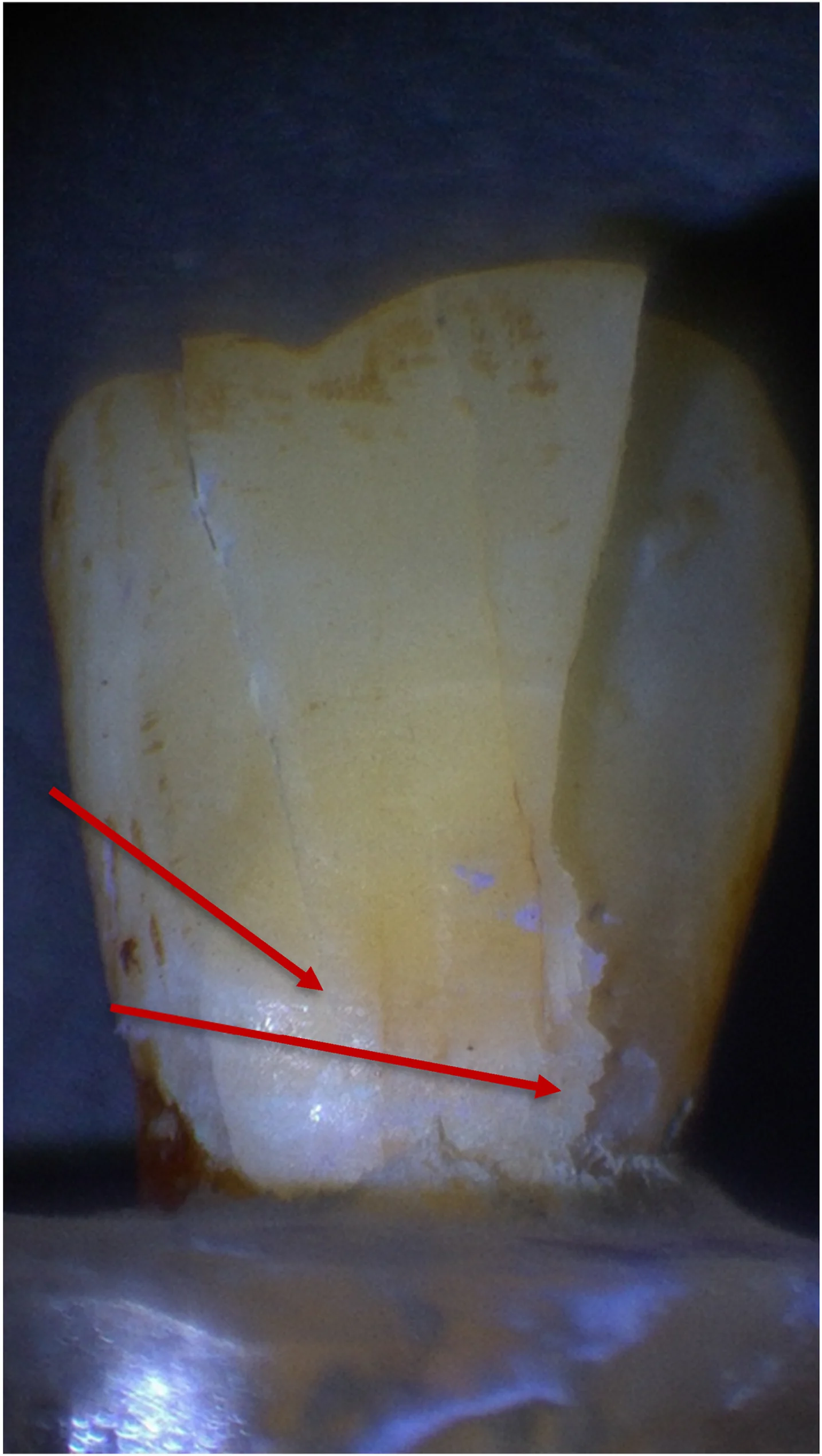
A stereomicroscope image with 8× magnification showing a favorable fracture pattern (above the CEJ). Arrows indicating the fracture site

The distribution of fracture patterns is presented in (Table 4) and illustrated in (Fig. 7). Representative fracture patterns are shown in (Figs. 8 and 9). Fisher’s Exact test demonstrated a statistically significant association between intra-canal restorative technique and fracture pattern (*P* = 0.014). Overall, 48 specimens (60%) exhibited favorable fractures, whereas 32 specimens (40%) showed unfavorable fractures. The highest percentage of favorable fractures was observed in the FS group, where all specimens (100%) demonstrated favorable failure. In contrast, the highest percentage of unfavorable fractures was observed in the DC groups, where 70% of specimens exhibited unfavorable fracture patterns. 

**Table 4:** Fracture pattern distribution frequency among different study groups

Study Group	Fracture pattern *n* (%)		*p*-value
	Favorable	Unfavorable	
PSI	6 (60%)	4 (40%)	0.014*
PSR	6 (60%)	4 (40%)	
FPI	5 (50%)	5 (50%)	
FPR	7 (70%)	3 (30%)	
FSI	8 (80%)	2 (20%)	
FSR	10 (100%)	0 (0%)	
DCI	3 (30%)	7 (70%)	
DCR	3 (30%)	7 (70%)	

*Statistically significant at *P* < 0.05

The reported p-value represents the comparison of fracture pattern distribution among all eight experimental subgroups using Fisher’s exact test

I: intact marginal ridge

R: removed marginal ridge

## Discussion

Endodontically treated anterior teeth are particularly susceptible to fracture because of the combined effects of tooth structure loss and unfavorable stress distribution under functional loading [1, 2]. Although several intra-canal reinforcement strategies have been proposed to improve their biomechanical behavior, evidence comparing short fiber-reinforced composites (SFRCs) with fiber posts in anterior teeth remains limited. Therefore, the present study evaluated the influence of different intra-canal restorative techniques on the fracture resistance and fracture patterns of endodontically treated anterior teeth.

Fiber posts were introduced to improve stress distribution because their modulus of elasticity approximates that of dentin [4, 16], thereby reducing stress concentration within the root structure. However, this concept remain controversial, and some authors suggest that fiber posts offer retentive functions rather than pure reinforcement [17]. This has led to more conservative restorative approaches by preserving the peri-cervical dentin to avoid excessive radicular dentin removal and using short fiber reinforced composite inside the canal instead of a traditional fiber post [6, 18].

Short fiber-reinforced composites (SFRCs) were developed to enhance fracture resistance and inhibit crack propagation through the incorporation of randomly oriented E-glass fibers within the resin matrix [7]. In the present study, both the packable (EverX Posterior) and flowable (EverX Flow) formulations (GC, Tokyo, Japan) were evaluated to determine whether differences in viscosity and handling characteristics influence their reinforcing potential. Conversely, dual cure core materials continue to be used due to their simplicity and cost-effectiveness, despite concerns regarding their reinforcing capacity [19].

From the results of this study, it was demonstrated that the type of intra-canal restorative technique significantly influenced the fracture resistance (p-value < 0.001; Table 2), so the first null hypothesis was rejected. The control group, included as a reference, exhibited the highest fracture resistance values, which is consistent with the general agreement that the main factor influencing mechanical durability is the amount of sound tooth structure, [20–22] as preserving the continuity of enamel and dentin allows optimal stress distribution. Among the restorative groups, the fiber post group demonstrated the highest values (Table 3). This could be explained by an even distribution of stresses within the root canal owing to a modulus of elasticity close to dentin, which reduces stress concentration at the cervical area [23] Similar outcomes have been reported by Jurema et al [24]. They found that fiber posts did improve fracture resistance of endodontically treated anterior teeth compared to alternative restorative treatments. However, Massafra et al. [25] evaluated glass fiber posts in endodontically treated teeth with and without Class III restorations and showed no significant statistical difference in fracture strength between any of the groups. This was attributed to the presence of sufficient tooth structure obviating the need for a fiber post. This is also consistent with other authors suggesting that fiber posts may offer a retentive function for a core in extensive tissue loss conditions rather than actual reinforcement [26, 27].

The SFRC groups showed no statistically significant difference in fracture resistance compared to fiber posts (*p* > 0.05). This may be attributed to the reinforcing effect of randomly distributed short E-glass fibers within the resin matrix, which exceed the critical fiber length (0.5–1.6 mm), enabling effective stress transfer and improved fracture toughness. Additionally, the presence of a semi-interpenetrating polymer network semi-IPN enhances mechanical performance [28]. These findings are consistent with Fráter Máté et al. [29], who reported increased fracture resistance using SFRC materials such as EverX Posterior and EverX Flow in anterior teeth. Similarly, Jain Vikas et al. [14] supported the use of SFRC as a viable intra-canal alternative to posts. However, this seems to go against Forster et al.‘s findings [30] that there was no significant difference in fracture resistance between teeth restored with a conventional Fiber post and those with packable SFRC inside the root canal. However, it should be noted that their study focused only on endodontically treated premolars with class I cavities.

Although packable SFRC exhibited slightly higher fracture resistance values than the flowable formulation, the difference was not statistically significant (Table 3). This finding may be explained by differences in the microstructural characteristics of the reinforcing glass fibers, particularly fiber length, diameter, and aspect ratio, which influence stress transfer from the polymer matrix to the fibers and improve resistance to crack propagation. EverX Posterior contains longer and thicker E-glass fibers than EverX Flow, which may contribute to its slightly greater reinforcing potential by promoting more effective stress distribution [31].

However, these findings contradict those of Fráter et al., [29] who reported superior mechanical performance of EverX Flow in certain restorative configurations. The discrepancy may be attributed to differences in the material composition of the two SFRC formulations, particularly fiber characteristics, as well as variations in the experimental design, including cavity configuration, material thickness, and loading conditions, all of which may influence stress distribution and fracture behavior. Similarly, Jain et al. [14]reported that intra-canal placement of EverX Flow in maxillary central incisors without post-space preparation resulted in fracture resistance comparable to that of intact teeth. It is noteworthy that their ultraconservative approach preserved the peri-cervical dentin, which may have contributed to the favorable mechanical performance. Collectively, these findings suggest that the effectiveness of SFRC restorations may be influenced by the amount of remaining tooth structure. However, further laboratory and clinical studies are required before recommending one restorative strategy over another in different clinical situations.

The dual-cure core material in this study exhibited the lowest fracture resistance values, which is consistent with literature indicating that conventional composite core materials lack fiber reinforcement and exhibit lower fracture toughness [32]. Stress concentration and catastrophic failures may be caused by polymerization shrinkage stresses and the absence of crack-deflecting mechanisms. Similar results were found in investigations where fracture resistance was not significantly increased by composite core materials [18, 33]. On the contrary, some authors reported that intra-canal dual-cure core composites, used either alone or in combination with fiber posts, can provide fracture resistance or fatigue survival comparable to fiber post or SFRC based techniques [29, 34]. This disagreement may be related to differences in tooth preparation, presence of crowns and ferrule which may have masked material-level differences by re-distributing stresses to the crown-ferrule complex, and the dual cure core material in these studies was combined with posts rather than used as an isolated intra-canal reinforcement material as in this study.

Regarding marginal ridge integrity, specimens with intact marginal ridges exhibited significantly higher fracture resistance values compared with those with loss of one marginal ridge (P-value < 0.001; Table 2). Therefore, the second null hypothesis was also rejected. This was attributed to the fact that the remaining coronal dentin wall thickness directly influenced fracture resistance in maxillary central incisors, where oblique loading generates high tensile stresses on the palatal surface. The loss of structural integrity through marginal ridge removal critically compromises their fracture resistance. These results are in agreement with several authors who reported that maxillary central incisors with four intact coronal walls exhibited significantly higher fracture resistance than those with only three walls, with palatal wall loss being particularly detrimental [35, 36]. In the present study, under both marginal ridge conditions, fiber posts, packable SFRC, and flowable SFRC demonstrated comparable fracture resistance with no statistically significant differences (Table 3). These findings are consistent with Bijelić-Donova et al. [37], who found no significant difference in fracture resistance of severely damaged maxillary incisors restored with fiber posts alone, short fiber composite with fiber posts, or short fiber composite used as a complete post-core-crown complex. Nonetheless, the similar outcomes of SFRC formulations with and without fiber posts reported by El Agamy [38] and Bijelić-Donova et al. imply that the biomimetic stress distribution offered by randomly orientated short fibers could compensate for the absence of a conventional post system, especially when enough coronal tooth structure is preserved, as in this study.

Regarding fracture patterns, 60% of the specimens had favorable fractures (Table 4). According to the fracture pattern analysis, this study demonstrated that the intra-canal restorative technique used had a statistically significant effect on the fracture patterns observed (*p* = 0.014). Notably, the EverX Flow group showed the highest percentage of favorable fractures, especially when the marginal ridge was lost, despite having lower overall fracture resistance values than the fiber post and EverX Posterior. These results are consistent with the “bioblock” concept described by Fráter et al. [39], according to which the coronal structure and the canal work together as a single reinforced complex. Improved adaptability inside narrow spaces like root canals, fewer internal voids, and a multidirectional fiber orientation may be responsible for the favorable fracture patterns seen even surpassing EverX Posterior. This finding is in agreement with Magne et al. [40]. They found that, while having a lower average strength, EverX Flow tended to show more repairable partial fractures than EverX Posterior. It was claimed that when EverX Posterior fails, its greater stiffness concentrates stress at the tooth-restoration interface and transfers greater load to the root. Because of EverX Flow’s lower elastic modulus, the restoration’s stress-absorbing layer fails through cohesive fracture or delamination before the root is damaged [41].

However, Guzmán Reyes et al. [42] showed conflicting data from anterior teeth without ferrule, stating that catastrophic failures were more prevalent in fiber-reinforced composite than in traditional fiber posts. This suggests that failure patterns may be influenced by particular clinical situations. The absence of ferrule in Guzman Reyes’ study creates a compromised foundation that may predispose fiber-reinforced composites to catastrophic failures due to inadequate stress distribution and lack of circumferential support.

Conversely, the dual-cure core material group exhibited the highest percentage of unfavorable fractures among study groups. Dual-cure composites are particulate-filled materials that lack any crack-stopping reinforcement, making them more prone to brittle, catastrophic failure than fiber-reinforced composites, which contain short randomly oriented E-glass fibers which can stop crack propagation through deflection mechanisms, [43] which is in agreement with the findings of several previous studies [37, 38, 44].

This study showed that the complex interaction between restorative technique, remaining tooth structure, and biomechanical compatibility determines fracture resistance and failure behavior of endodontically treated anterior teeth. Fiber-reinforced intra-canal approaches demonstrated improved stress distribution and a higher tendency toward favorable fracture patterns. From a clinical perspective, the comparable behavior of short fiber-reinforced composites to conventional fiber posts further suggests their potential as a post-free alternative. Intra-canal placement of SFRC extending 4 mm apical to the CEJ may contribute to reducing the tensile stresses concentrated at this fulcrum region. These findings should be confirmed through long-term clinical and fatigue-loading studies before routine clinical implementation. However, fiber posts may remain the preferred restorative option in teeth with extensive coronal destruction or compromised structural support, where additional retention is required. Nevertheless, treatment selection should be individualized according to the remaining tooth structure.

One limitation of this study is that it was carried out in vitro, which is unable to accurately replicate the complex biological and mechanical environment of the oral cavity. The long-term clinical performance of restorative materials may be impacted by clinical variables such as saliva, occlusal dynamics, periodontal ligament response, and patient-related factors. Static loading to failure was used to assess fracture resistance. However, this method does not adequately mimic the cyclic fatigue stresses produced during mastication, as crack propagation usually happens gradually under repeated loading as opposed to a single catastrophic force. Although thermocycling was performed to simulate thermal changes in the oral environment, the aging protocol represented only a limited period of clinical service and may not reflect long-term intraoral degradation. The periodontal ligament was not simulated during testing, which may influence stress distribution during loading. Therefore, the teeth were embedded directly in auto polymerizing acrylic resin to ensure standardized specimen fixation and reproducible mechanical testing conditions. Although simulation of the periodontal ligament using silicone-based materials has been recommended to better reproduce physiological tooth mobility, its influence on fracture resistance testing remains controversial, with previous studies reporting conflicting findings regarding its effect on fracture resistance values and fracture patterns [45, 46]. Therefore, further studies incorporating cyclic loading protocols, long-term aging procedures, and clinical trials are recommended to better validate the results of the present investigation.

## Conclusions

Within the limitations of this in vitro study, the intra-canal restorative technique significantly influenced the fracture resistance and fracture pattern of endodontically treated anterior teeth. Fiber post restorations showed the highest numerical fracture resistance values, while short fiber-reinforced composite restorations showed fracture resistance values that were not statistically different from fiber posts and were associated with more favorable fracture patterns. Loss of one marginal ridge significantly reduced fracture resistance, highlighting the importance of preserving coronal tooth structure. Further studies using fatigue loading, periodontal ligament simulation, and long-term aging protocols are required before clinical recommendations can be made.

## Data Availability

The datasets used and/or analyzed during the current study are available from the corresponding author on reasonable request.
